# Design of Long-Wave Fully Polarized HgCdTe Photodetector Based on Silicon Metasurface

**DOI:** 10.3390/mi16080937

**Published:** 2025-08-14

**Authors:** Bo Cheng, Xiaoming Wang, Yuxiao Zou, Guofeng Song, Kunpeng Zhai, Xiaojun Wang

**Affiliations:** 1 Postdoctoral Innovation Practice Base, Chengdu Polytechnic, 83 Tianyi Street, Chengdu 610041, China; chengbo9610@semi.ac.cn (B.C.); ebianwxm@gmail.com (X.W.); 2 Sichuan Provincial Engineering Research Center of Thermoelectric Materials and Devices, Chengdu 610041, China; 3 Kunming Institute of Physics, Kunming 650223, China; 13307110322@fudan.edu.cn; 4 Institute of Semiconductors, Chinese Academy of Sciences, Beijing 100083, China; sgf@semi.ac.cn; 5 Institute of Intelligent Photonics, Nankai University, Tianjin 300071, China

**Keywords:** metasurface, polarization-sensitive photodetection, extinction ratio

## Abstract

Polarization-sensitive photodetection is critical for advanced optical systems, yet achieving simultaneous high-fidelity recognition of the circularly polarized (CP) and linearly polarized (LP) light with compact designs remains challenging. Here, we use COMSOL 5.6 software to demonstrate a silicon metasurface-integrated MCT photodetector that resolves both CP and LP signals through a single ultrathin platform. The device deciphers LP states via four orientation-specific linear gratings for differential detection, while chiral symmetric silicon nanostructures enable direct CP discrimination with an exceptional extinction ratio of 30 dB. The proposed architecture combines two breakthroughs: (1) superior polarization reconstruction capability, achieved via the synergy of grating-induced polarization selectivity and chiral near-field enhancement, and (2) a fabrication-simplified process that eliminates multilayer stacking or complex alignment steps. This work establishes a new paradigm for miniaturized, high-performance polarization optics, with potential applications in polarization imaging, quantum communication, and hyperspectral sensing.

## 1. Introduction

Long-wave infrared photodetectors based on mercury cadmium telluride (MCT) [1] are crucial for applications such as molecular spectroscopy, thermal imaging, and medical diagnosis [2,3]. However, traditional MCT detectors themselves cannot distinguish the polarization state of photons. In scenarios such as chiral molecule detection [4] or polarization thermal radiation analysis [5], the polarization state carries indispensable information. Although linear polarizers [6,7,8] (such as linear grating structures) can be combined with MCT detectors to extract linear polarization states, research on how to obtain complete Stokes parameters, including circular polarization (CP), in a single chip device is still scarce.

Recent advancements in chiral metasurfaces [9,10,11,12,13] have made it possible to directly detect circularly polarized light through selective transmission or near-field electric field enhancement under left or right circularly polarized (LCP/RCP) illumination. However, most designs only operate in the visible [14,15,16,17,18,19,20] or near-infrared bands [21,22,23,24,25,26,27,28,29], and the designs in the longer wavelength region are relatively scarce [30,31,32,33]. This is mainly due to challenges in material loss and manufacturing scalability [34,35,36]. Additionally, existing long-wave infrared circular polarization-sensitive detectors typically require complex heterostructures or huge external optical components, increasing system complexity and limiting integration potential.

In this work, we designed a chiral MCT detector capable of recognizing circularly polarized light. Its fabrication process is quite simple, requiring only the growth of a silicon thin film followed by one photolithography. We also investigated the electric field distribution of the silicon metasurface and discovered remarkable local electric field enhancement phenomena, which correspond to the transmission minima of RCP and the transmission maxima of LCP. Additionally, we analyzed the influence of the linear grating on the MCT and the potential performance degradation of the micro-device due to process errors.

## 2. Materials and Methods

The Stokes parameters (S_1_, S_2_, S_3_, S_4_) can fully describe the polarization information of light. The first three elements are closely related to linear polarization information, while the fourth element represents circular polarization signals. According to the view of reference [37], to achieve the complete collection of Stokes parameters, six independent polarization pixels are required, which respectively only absorb the 0-degree linearly polarized light signal $I_{0}$, 90-degree linearly polarized light signal $I_{90}$, 45-degree linearly polarized light signal $I_{45}$, 135-degree linearly polarized light signal $I_{135}$, left circularly polarized light signal $I_{LCP}$, and right circularly polarized light signal $I_{RCP}$ in spatial light. The Stokes parameters can be restored through the following formula:

(1) $$S_{0}=I_{0}+I_{90}$$

(2) $$S_{1}=I_{0}-I_{90}$$

(3) $$S_{2}=I_{45}-I_{135}$$

(4) $$S_{3}=I_{LCP}-I_{RCP}$$

When using the technical solution of metasurfaces combined with photodetectors to achieve polarization detection, six metasurfaces with polarization filtering functions are required. Their transmission rates for specific polarized light should be close to zero, while they have considerable transmission rates for the orthogonal polarized light. Generally, there are two performance indicators for the filter plates in polarization detectors. The first one is the circular dichroism (CD), which is the difference in transmission rates of these two orthogonal modes. The higher it is, the more incident light can be converted into electron–hole pairs (or photocurrent). The second one is the extinction ratio (RE), which is the ratio of the transmission rates of these two orthogonal modes or the logarithm of the ratio. The larger it is, the stronger the theoretical ability of Formulas (1)–(4) to restore the polarization signal.

**Figure 1:**
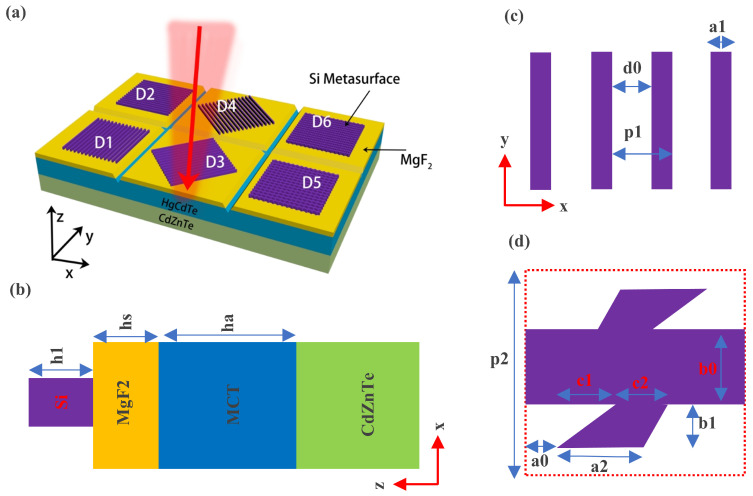
Fully polarized MCT detector. (**a**): Three-dimensional structure diagram of circularly polarized detector and linearly polarized detector. D1, D2, D3, D4, D5, D6 can, respectively, respond to 90-degree linearly polarized light, 0-degree linearly polarized light, 45-degree linearly polarized light, 135-degree linearly polarized light, as well as left circularly polarized light and right circularly polarized light. The six metasurfaces correspond to six independent detector pixels. The polarization detector is isolated through the mesa junction technology to ensure physical separation between the pixels. (**b**) Front view of linearly polarized detector (D1). ha = 10 μm, hs = 4.4 μm, h1 = 3.65 μm. (**c**) xy cross-sectional view of the grating in the linearly polarized detector. p1 = 5.56 μm, d0 = 3.9 μm. (**d**) xy cross-sectional view of the silicon metasurface in the circularly polarized detector. p2 = 5.2 μm, a0 = 0.55 μm, a2 = 1.77 μm, b0 = 1.79 μm, b1 = 1.13 μm, c1 = 0.32 μm, c2 = 2.2 μm.

Figure 1a shows the three-dimensional structure of the fully polarized MCT detector, which is composed of a 0-degree linear polarization detector, a 90-degree linear polarization detector, a 45-degree linear polarization detector, a 135-degree linear polarization detector, and left and right circular polarization detectors. Their fabrication process is very simple, requiring only the growth of 3.65 μm of amorphous silicon on the anti-reflection film (MgF2) of a conventional MCT, followed by one photolithography process. The structure of the gratings in the four linear polarization detectors is the same, but their long axes are oriented differently. The silicon metasurfaces in the two circular polarization detectors are mirror-symmetric about the yz plane. Figure 1b is a front view of the 0-degree linear polarization detector, which provides information on the material composition and thickness of the polarization detector. Figure 1c shows the xy cross-section of the linear grating in the 0-degree linear polarization detector, which contains the structure information of the silicon grating. Figure 1d shows the xy cross-section of the unit cell of the chiral metasurface in the left circular polarization detector, which is composed of a rectangle at the center and two quadrilaterals above and below. The upper quadrilateral can be obtained by rotating the lower quadrilateral 180 degrees around the center position. The optical refractive index of the CdZnTe substrate is 2.68, and the refractive index parameters of MCT are from reference [38]. The refractive indices of MgF2 and silicon are 1.38 and 3.48, respectively. The absorption rate of the polarization detector is calculated based on COMSOL software. Periodic boundary conditions are used on the four sides of the optical model to simulate an infinite periodic structure and ensure the continuity of the field between adjacent units. Perfectly matched layer (PML) conditions are used on the upper and lower interfaces of the optical model to absorb outgoing waves and simulate an open boundary with no reflection truncation. The incident port is approximately one wavelength away from the metasurface and can be used to set the initial phase, direction, and polarization of the incident light. The reflectance and transmittance can be obtained by integrating the Poynting vector on the upper and lower boundaries of the model, and the optical absorption rate of the polarization detector is 1 minus the reflectance minus the transmittance.

## 3. Results

**Figure 2:**
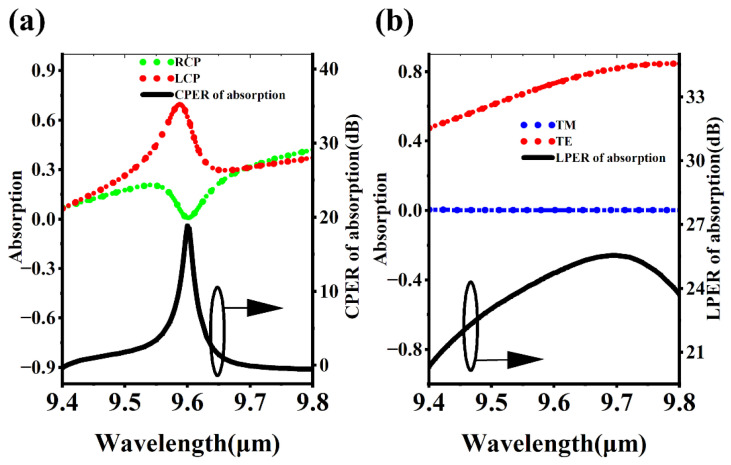
Spectra of polarization detectors. (**a**) Absorption spectrum and CPER spectrum of circular polarization detector. (**b**) Absorption spectrum and LPER spectrum of linear polarization detector. The curves marked with arrows should correspond to the right *Y*-axis, not the left one.

The core components of the six-in-one full polarization MCT detector are the 90-degree linearly polarized MCT and left circularly polarized MCT. The other polarization pixels can be obtained by performing geometric operations on them. Figure 2a shows the absorption of the left circularly polarized MCT for RCP and LCP. It can be found that for RCP, the absorption increases first, then decreases, and then increases again in the 9.4 to 9.8 μm band, with the minimum absorption occurring at a wavelength of 9.6 μm. The maximum absorption peak of LCP also appears near 9.6 μm, with an absorption peak value close to 0.7. The circular polarization extinction ratio (CPER) of the left circularly polarized MCT is 10 $\times log(A_{LCP}/A_{RCP})$, where $A_{LCP}$ and $A_{RCP}$ are the absorption of the polarization pixels for LCP and RCP, respectively. The CPER spectrum is a typical sharp narrow peak, with a peak wavelength of 9.6 μm and a peak intensity of approximately 19 dB. Figure 2b shows the absorption spectrum and linear polarization extinction ratio (LPER) spectrum of the 90-degree linearly polarized MCT. LPER = 10 $\times log(A_{TE}/A_{TM})$, where $A_{TE}$ and $A_{TM}$ are the absorption of the polarization detector for TE and TM, respectively. TE and TM correspond to the cases where the electric field amplitude of the linearly polarized light is in the y and x directions, respectively. For the TM mode, the absorption is close to zero throughout the 9.4 to 9.8 μm band, showing a strong broadband effect. For the TE mode, the absorption increases with the increase in wavelength, with an average absorption rate greater than 0.6. The maximum LPER value occurs at a wavelength of 9.68 μm, approximately 25.5 dB. The LPER at 9.6 μm wavelength is approximately 24 dB.

### 3.1. The Polarization Discrimination Capability of the Linear Grating

**Figure 3:**
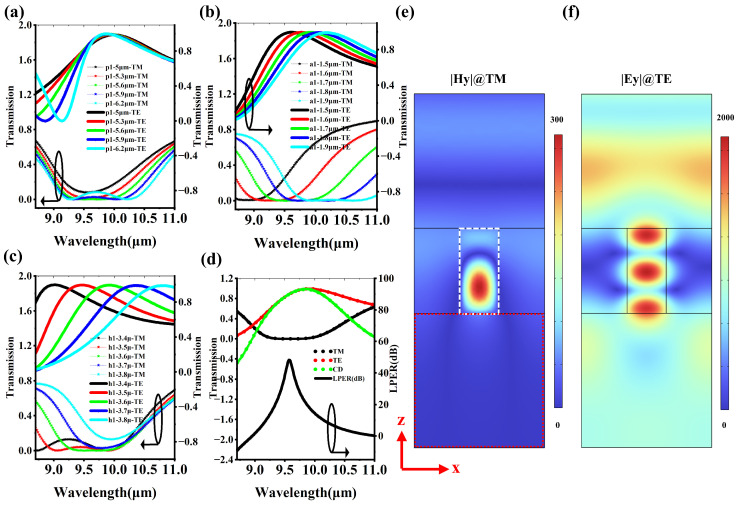
Transmission spectra of the linear grating. (**a**) The influence of the grating period p1. (**b**) The influence of the grating width a1. (**c**) The influence of the grating height h1. (**d**) Transmission spectra and LPER spectra of the line grating. (**e**) Magnetic field distribution in the TM mode. (**f**) Electric field distribution in the TE mode. The white dashed box marks the boundary of the silicon pillar.

It should be emphasized that the MCT detector itself cannot distinguish polarized signals; it requires the assistance of the polarizing grating on its top. For the 90° linearly polarized MCT, the TE mode can penetrate the linear grating and enter the light absorption area of the MCT to form the photoelectric current, while for the TM mode, the linear grating exhibits the function of an optical mirror, preventing photons from passing through it to reach the light absorption area of the MCT. Figure 3a–c show the influence of the grating’s structural parameters on the transmission spectrum. As shown in Figure 3a, with the increase in the period *p1*, the transmission rate at a wavelength of 9.6 μm changes very little. Especially for the TM mode, when the wavelength is greater than 10 μm, the absorption spectra are almost identical. Figure 3b indicates the transmission spectrum redshift as the grating width *a1* increases. If the grating width *a1* is increased by just 0.4 μm, the transmission peak of the TE mode can be shifted from 9 μm to 10.5 μm. Figure 3c shows that the transmission spectra of both TE and TM modes exhibit redshift as the grating thickness *h1* increases, but the intensity of the spectral line shift of the TE mode is far more intense than that of the TM mode. Figure 3d presents the transmission spectrum and the linear polarization extinction ratio (LPER) spectrum of the optimized grating. Its maximum CD occurs at a wavelength of approximately 9.8 μm, with a peak close to 1. However, the LPER peak is at a wavelength of 9.6 μm, with a peak value of about 50 dB. Here, LPER represents the grating’s extreme suppression effect on the TM mode, the LPER is 10 $\times log(T_{TE}/T_{TM})$, where $T_{TE}$ and $T_{TM}$ are the transmission of the linear grating for TE and TM, respectively. Figure 3e,f show the near-field electric field intensity distribution of the grating. For the TM mode at a wavelength of 9.6 μm, a large red sphere appears inside the silicon grating, which satisfies the typical characteristics of a standing wave. This standing wave exhibits a typical photonic bandgap effect, with almost no free photons being able to radiate into the medium below. For the TE mode at a wavelength of 9.6 μm, the electric field localization sphere in the silicon grating shows a radiative state mode, and it can be found that some photons can leak laterally into the air domain on the side. This radiative state allows the photon energy to penetrate the silicon grating and enter the MgF_2_ below.

### 3.2. The Circular Dichroism Analysis of Chiral Metasurfaces

**Figure 4:**
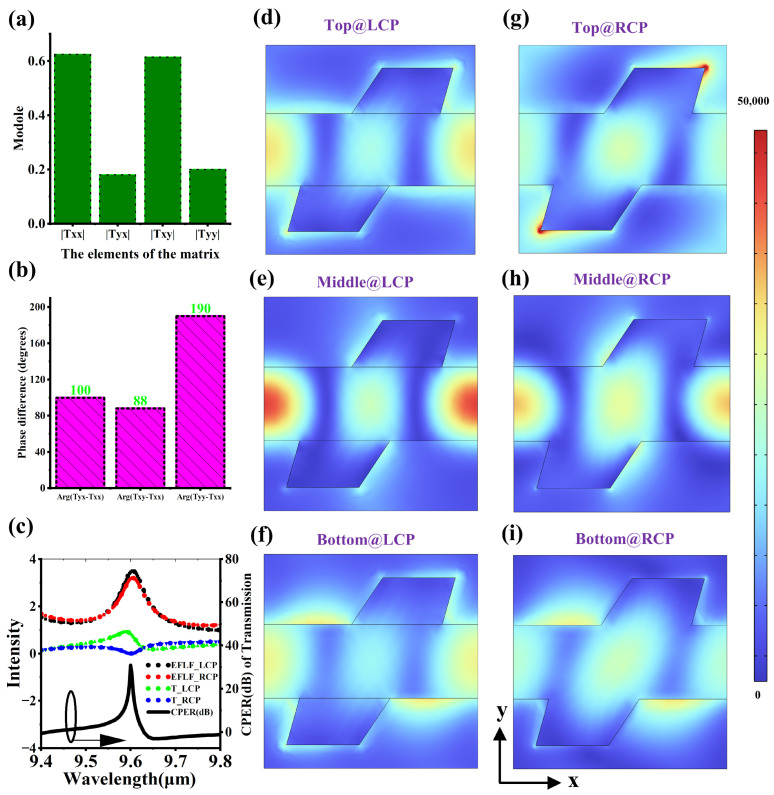
Optical mode analysis of chiral metasurfaces. (**a**) The modulus of the elements of the Jones matrix corresponding to the chiral metasurface. (**b**) The phase difference between the elements of the Jones matrix. (**c**) The EFLF spectrum, transmission spectrum, and CPER spectrum of the chiral metasurface. (**d**–**i**) Electric field distribution maps of the chiral metasurface in CP mode. “Top”, “Middle”, and “Bottom”, respectively, indicate that the cross-section is located at the top, middle, and bottom of the metasurface.

Figure 4a,b, respectively, show the amplitude and phase of the elements of the Jones matrix of the chiral metasurface in left circularly polarized MCT, which can describe the transmission characteristics of the chiral metasurface. It can be found that |Txx| = |Txy| = a, |Tyx| = |Tyy| = b, arg(Tyx − Txx) is approximately 90°, arg(Txy − Txx) is approximately 90°, and arg(Tyy − Txx) is approximately 180°. Therefore, the Jones matrix of the chiral metasurface can be simplified as T = $\left[\begin{array}{cc} a & a*i\\ b*i & -b \end{array}\right]$. Since T $\times \frac{\sqrt{2}}{2}[\begin{array}{c} 1\\ i \end{array}]$ = 0 $\times \frac{\sqrt{2}}{2}[\begin{array}{c} 1\\ i \end{array}]$, T $\times \frac{\sqrt{2}}{2}[\begin{array}{c} 1\\ -i \end{array}]$ = 2 $\times [\begin{array}{c} a\\ b*i \end{array}]$, where $\frac{\sqrt{2}}{2}[\begin{array}{c} 1\\ i \end{array}]$ and $\frac{\sqrt{2}}{2}[\begin{array}{c} 1\\ -i \end{array}]$ correspond to the Jones vectors of RCP and LCP, respectively. The eigenvalue of 0 in the RCP case explains why the transmission rate of RCP is close to 0. Figure 4c shows the electric field localization factor spectrum (EFLF), transmission spectrum, and CPER spectrum of the chiral metasurface. EFLF can represent the strength of the electric field localization, which $\int |E|dV/\int |E_{0}|dV$, where $E_{0}$ is the electric field intensity of the incident light and E is the electric field inside the silicon metasurface. The integration region is the volume of the silicon metasurface. It can be found that the EFLF of RCP and LCP both have a maximum value near 9.6 μm. Within the wavelength range of 9.4 to 9.8 μm, the maximum transmission of LCP is close to 0.9, the minimum transmission of RCP is close to 0, and the maximum CPER is close to 30 dB. Here, CPER is mainly used to measure the difference in the transmission performance of CP, and it is defined as $T_{LCP}/T_{RCP}$, where $T_{LCP}$ and $T_{RCP}$ are the transmissions of the metasurface for TE and TM, respectively. Figure 4d–i show the electric field distribution diagrams of the cross-section of the metasurface. It can be found that there are strong electric field localization effects at different cross-sections under different CP modes, which often correspond to the electromagnetic resonances supported by the nanostructures. We boldly speculate that in the RCP incident mode, the incident field and the scattering field formed by the metasurface acting as electromagnetic radiation sources form a phenomenon of destructive interference, cancelling each other out at the transmission end, resulting in a minimum transmission rate. In the LCP incident mode, the incident light and the transmitted electromagnetic field excited by the metasurface form a coherent constructive interference effect, contributing to the formation of the LCP transmission peak.

### 3.3. The Parameter Optimization of the Spacer Layer hs

**Figure 5:**
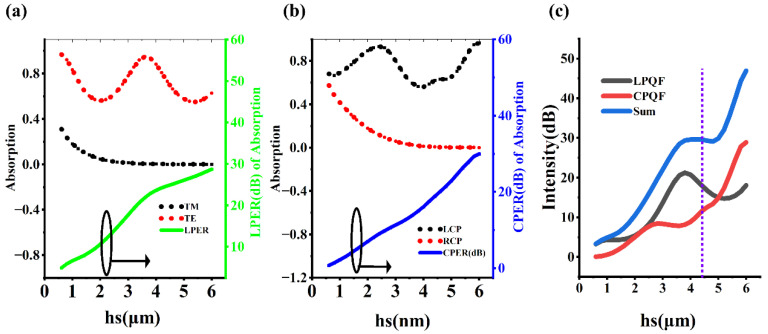
The influence of the thickness *hs* between the metasurface and MCT. (**a**) The influence of *hs* on the absorption and LPER of the linearly polarized MCT. (**b**) The influence of *hs* on the absorption and CPER of the circularly polarized MCT. (**c**) LPQF and CPQF related to *hs*.

In the previous section, we emphasized that the polarization discrimination ability stems from the effective screening of different polarized lights by the metasurface, rather than the anisotropic absorption of MCT itself. In addition, to transfer the polarization discrimination ability of the metasurface entirely to the light absorption region of MCT, it is necessary to make the metasurface and MCT two independent optical components as much as possible, which helps to avoid the performance degradation caused by the optical coupling between them. Generally, in the optical field, we can optimize the coupling effect by adjusting the distance between two optical components. Figure 5a shows the influence of the distance *hs* between the metasurface and MCT on the polarization discrimination ability of the linearly polarized detector. The absorption of TM decreases with the increase in *hs*. When *hs* is greater than 3 μm, the absorption remains at a very low and stable level. The absorption of TE shows a cosine function spectrum with the increase in *hs*, which is a regular periodic vibration. In addition, LPER gradually increases with the increase in *hs*. Figure 5b shows the influence of the distance *hs* on the circularly polarized MCT, and its rule is very similar to that in Figure 5a. It is necessary to note an unavoidable phenomenon that the extinction ratio and CD do not increase simultaneously with the increase in *hs*. Therefore, a new comprehensive performance index for measuring the performance of the polarization detector, the polarization quality factor (PQF), is proposed. It is equal to the sum of the linear polarization quality factor (LPQF) and the circular polarization quality factor (CPQF), where LPQF = CD × LPER and CPQF = CD × CPER, which are used to measure the polarization selection ability of the linearly polarized MCT and the circularly polarized MCT, respectively. When *hs* is less than 3 μm, PQF, LPQF, and CPQF gradually increase with the increase in *hs*. When *hs* is greater than 3 μm, LPQF first increases and then decreases, CPQF first slightly decreases and then continuously increases, and PQF first increases, then remains unchanged, and then continues to increase. Obviously, when *hs* is equal to 6 μm, all three indicators are optimal values. However, considering the difficulty of growing a 6 μm thick MgF2 film, ultimately, *hs* = 4.4 μm is selected as the most appropriate value, as it corresponds to a relatively high PQF value and also represents a manageable difficulty in thin-film growth.

### 3.4. The Analysis of Potential Errors

**Figure 6:**
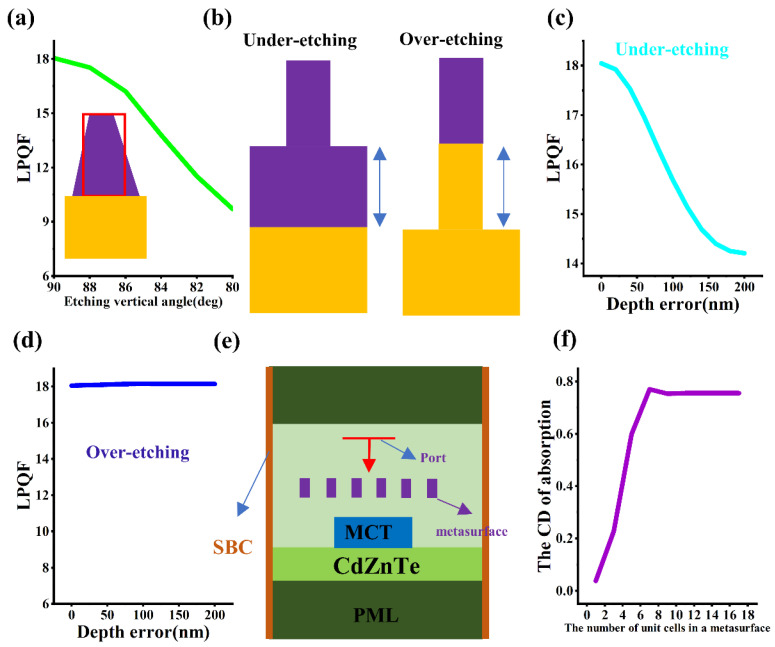
Error analysis of the linearly polarized MCT. (**a**) Influence of etching tilt angle. (**b**) Geometric diagrams of the under-etching and over-etching cases. (**c**) Influence of the under-etching depth error. (**d**) Influence of the over-etching depth error. (**e**) Schematic diagram of a two-dimensional simulation model for analyzing the number of unit cells of metasurface. (**f**) Influence of the number of unit cells of metasurface.

In the actual fabrication process of metasurfaces, many process errors can cause the structure of the metasurface to deviate from the ideal value, ultimately resulting in performance loss. After photolithography and etching, the rectangular structure usually turns into a trapezoid due to the difficulty in ensuring 100% anisotropy in the process, as shown in the inset of Figure 6a. Figure 6a shows the influence of the etching tilt angle (i.e., the lower base angle of the trapezoid) on the performance of the linearly polarized MCT. It can be found that as the etching tilt angle decreases, the LPQF also decreases slowly. When the etching tilt angle is 80 degrees, the LPQF is reduced by half. Figure 6b shows the under-etching and over-etching situations of the silicon metasurface, indicating the etching residue of the silicon material. Figure 6c shows that when the under-etching depth error increases from 0 nm to 200 nm, the LPQF decreases from 18 dB to 14 dB. Figure 6d shows that the over-etching depth error has almost no effect on the LPQF value. Figure 6e shows a two-dimensional optical simulation model for calculating the number of unit cells of the linear grating, with perfect matching layer conditions applied to the upper and lower end faces and scattering boundary conditions (SBC) applied to the left and right end faces. As shown in Figure 6f, as the number of unit cells increases, the dichroism of the linearly polarized detector first increases and then remains unchanged, with the inflection point corresponding to approximately six unit cells.

**Figure 7:**
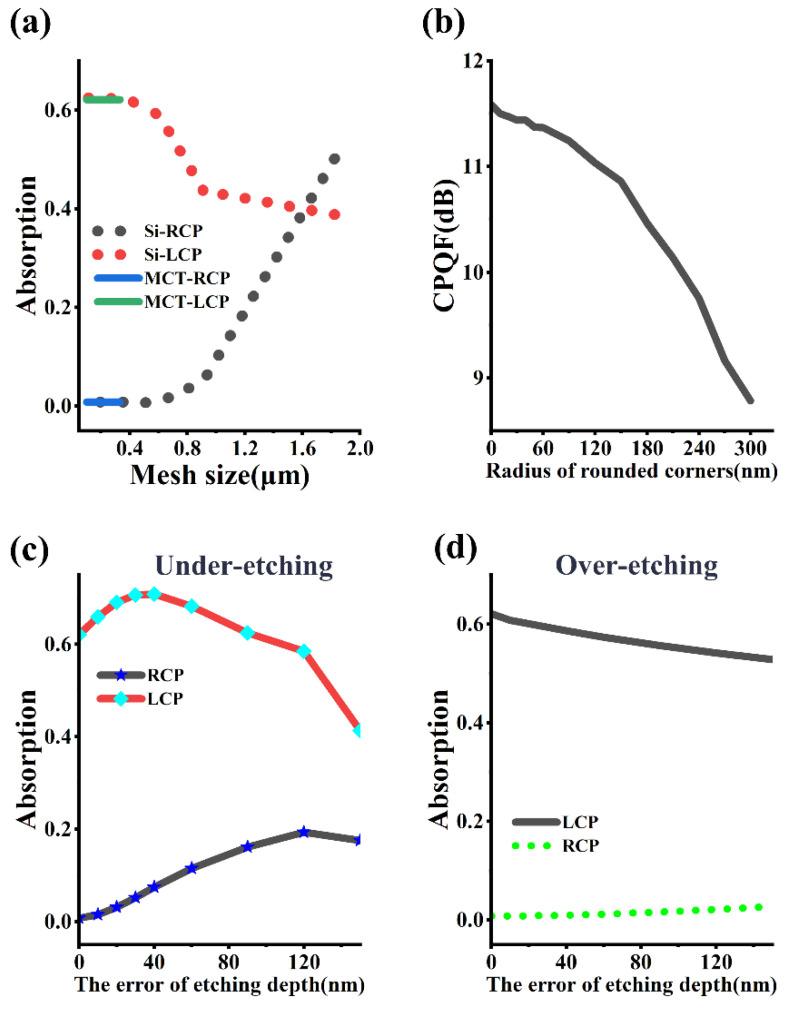
Error analysis of circularly polarized MCT. (**a**) The influence of the grid size. (**b**) The influence of the filet radius. (**c**) The influence of under-etching depth error. (**d**) The influence of over-etching depth error.

Figure 7 illustrates the influence of possible errors in the circularly polarized MCT. Figure 7a shows the impact of the grid size of the absorption region in the MCT and the grid size of the silicon metasurface. The variation in the MCT grid size within the range of 30 nanometers to 400 nanometers does not have a significant effect on the absorption of circularly polarized light. When the grid size of the silicon metasurface exceeds 0.5 μm, the absorption of LCP drops sharply, while that of RCP increases rapidly. Additionally, due to process factors such as photoresist reflow, insufficient anisotropy during etching, ion bombardment, or thermal diffusion effects, edge smoothing occurs, causing rectangular structures to become rounded (e.g., the edges of grooves or patterns become rounded); this special rounded corner phenomenon cannot be ignored either. Figure 7b shows the degree of damage to the performance of the circularly polarized detector caused by the radius of the etched round corners. When the radius of the etched round corners increases from 0 nm to 300 nm, the CPQF decreases from 11.5 dB to 9 dB. As shown in Figure 7c, with the increase in the under-etching depth error, the absorption of LCP first increases and then decreases, while that of RCP keeps increasing. When the etching depth error is 140 nm, the circular dichroism has already become very low. Figure 7d demonstrates the impact of over-etching. Although the error in the over-etching depth also has an adverse effect on the circularly polarized detector, its degree of damage is far less than that of under-etching. Given this situation, when etching the silicon metasurface, the etching time can be increased by a few seconds to ensure that the etching is in a critical etching or over-etching state. In addition, the influence of the incident angle and the MgF_2_ film on the micro-device can be seen in the Supplementary Materials.

## 4. Discussion

In summary, we demonstrate a monolithic MCT photodetector with circular polarization resolution capability at a wavelength of 9.6 μm, which is achieved by directly integrating a Si metasurface with ultra-large chiral transmission difference. The detector has a huge polarization extinction ratio of 19 dB for LCP and RCP. Crucially, when combined with a linearly polarized MCT based on a wire grating, our six-in-one device forms a compact photonic system capable of enabling full polarization reconstruction without the need for bulky optical components. This design bridges the gap between polarization optics and semiconductor photodetection, providing a scalable solution for miniaturized long-wave infrared polarization imaging and sensing applications.

## Data Availability

The raw data supporting the conclusions of this article will be made available by the authors on request.

## Supplementary Materials

The following supporting information can be downloaded at: https://www.mdpi.com/article/10.3390/mi16080937/s1, Figure S1. The influence of the incident angle. (a) The incident plane is the XZ plane. (b) The incident plane is the YZ plane. Figure S2. The influence of the changes in thickness and refractive index of the spacer layer caused by the temperature effect. (a) The variation in the thickness of the spacer layer ∆hs. (b) The variation in the refractive index of the spacer layer ∆n. Table S1: Meshing strategy of the model.

## References

[1] Rogalski A. (2005). HgCdTe infrared detector material: History, status and outlook. Rep. Prog. Phys..

[2] Eich D., Schirmacher W., Hanna S., Mahlein K.M., Fries P., Figgemeier H. (2017). Progress of MCT Detector Technology at AIM Towards Smaller Pitch and Lower Dark Current. J. Electron. Mater..

[3] Marcott C., Reeder R.C. (1998). FT-IR spectroscopic imaging microscopy using an MCT focal-plane array detector. Am. Inst. Phys..

[4] Anet F.A.L., Kopelevich M. (1989). Detection and assignments of diastereotopic chemical shifts in partially deuteriated methyl groups of a chiral molecule. Cheminform.

[5] Troitsky A.V., Osharin A.M., Korolev A.V., Strapp J.W. (2003). Polarization of Thermal Microwave Atmospheric Radiation Due to Scattering by Ice Particles in Clouds. J. Atmos. Sci..

[6] Deng Y., Meng C., Thrane P.C.V., Sande S.I., Bozhevolnyi S.I., Ding F. (2024). MEMS-integrated metasurfaces for dynamic linear polarizers. Optica.

[7] Han C., Tam W.Y. (2015). Plasmonic ultra-broadband polarizers based on Ag nano wire-slit arrays. Appl. Phys. Lett..

[8] Cui Y., Azzam R.M.A. (1996). Sixteen-beam grating-based division-of-amplitude photopolarimeter. Opt. Lett..

[9] Wu C., Arju N., Fan J., Brener I., Shvets G. Spectrally selective chiral silicon metasurfaces based on infrared Fano resonances. Proceedings of the Lasers & Electro-optics.

[10] Li Z., Liu W., Cheng H., Chen S., Tian J. (2016). Tunable dual-band asymmetric transmission for circularly polarized waves with graphene planar chiral metasurfaces. Opt. Lett..

[11] Zhang N., Gao F., Wang R., Shen Z., Han D., Cui Y., Zhang L., Chang C., Qiu C.W., Chen X. (2025). Deep-Learning Empowered Customized Chiral Metasurface for Calibration-Free Biosensing. Adv. Mater..

[12] Gromyko D., Loh J.S., Feng J., Qiu C.W., Wu L. (2025). Enabling All-to-Circular Polarization Up-Conversion by Nonlinear Chiral Metasurfaces with Rotational Symmetry. Phys. Rev. Lett..

[13] Ma C., Yu P., Jing Z., Zhu Y., Li P., Wang W., Xu H., Zhang Y., Pan L., Choi T.-Y. (2024). Circular polarization-selective optical, photothermal, and optofluidic effects in chiral metasurfaces. Photonics Res..

[14] Zhao Y., Belkin M.A., Alù A. (2012). Twisted optical metamaterials for planarized ultrathin broadband circular polarizers. Nat. Commun..

[15] Dietrich K., Menzel C., Lehr D., Puffky O., Huebner U., Pertsch T., Tuennermann A., Kley E.B. (2014). Elevating optical activity: Efficient on-edge lithography of three-dimensional starfish metamaterial. Appl. Phys. Lett..

[16] Bachman K.A., Peltzer J.J., Flammer P.D., Furtak T.E., Collins R.T., Hollingsworth R.E. (2012). Spiral plasmonic nanoantennas as circular polarization transmission filters. Opt. Express.

[17] Fedotov V.A., Schwanecke A.S., Zheludev N.I., Khardikov V.V., Prosvirnin S.L. (2007). Asymmetric Transmission of Light and Enantiomerically Sensitive Plasmon Resonance in Planar Chiral Nanostructures. Nano Lett..

[18] Cadusch J.J., James T.D., Djalalian-Assl A., Davis T.J., Roberts A. (2014). A Chiral Plasmonic Metasurface Circular Polarization Filter. IEEE Photonics Technol. Lett..

[19] Zuo J., Bai J., Choi S., Basiri A., Chen X., Wang C., Yao Y. (2023). Chip-integrated metasurface full-Stokes polarimetric imaging sensor. Light Sci. Appl..

[20] Li L.W., Rubin N.A., Juhl M., Park J.-S., Capasso F. (2023). Evaluation and characterization of imaging polarimetry through metasurface polarization gratings. Appl. Opt..

[21] Li W., Coppens Z.J., Besteiro L.V., Wang W., Govorov A.O., Valentine J. (2015). Circularly polarized light detection with hot electrons in chiral plasmonic metamaterials. Nat. Commun..

[22] Maksimov A.A., Tartakovskii I.I., Filatov E.V., Lobanov S.V., Gippius N.A., Tikhodeev S.G., Schneider C., Kamp M., Maier S., Höfling S. (2014). Circularly polarized light emission from chiral spatially-structured planar semiconductor microcavities. Phys. Rev. B.

[23] Lobanov S.V., Weiss T., Gippius N.A., Tikhodeev S.G., Kulakovskii V.D., Konishi K., Kuwata-Gonokami M. (2015). Polarization control of quantum dot emission by chiral photonic crystal slabs. Opt. Lett..

[24] Lobanov S.V., Tikhodeev S.G., Gippius N.A., Maksimov A.A., Hfling S. (2015). Controlling circular polarization of light emitted by quantum dots using chiral photonic crystal slab. Phys. Rev. B.

[25] Basiri A., Chen X., Bai J., Amrollahi P., Carpenter J., Holman Z., Wang C., Yao Y. (2019). Nature-inspired chiral metasurfaces for circular polarization detection and full-Stokes polarimetric measurements. Light. Sci. Appl..

[26] Hu J., Zhang C., Dong Y., Zeng A., Wang C. (2021). High efficiency all-dielectric pixelated metasurface for near-infrared full-Stokes polarization detection. Photonics Res..

[27] Cheng B., Zou Y., Shao H., Li T., Song G. (2020). Full-Stokes imaging polarimetry based on a metallic metasurface. Opt. Express.

[28] Cheng B., Zou Y., Song G. (2025). Ultra-large bandwidth fully polarized photodetectors based on displacement-induced chiral dielectric metasurfaces. Appl. Opt..

[29] Cheng B., Zou Y., Song G. (2024). Full-stokes polarization photodetector based on the chiral metasurface with the dislocated double gold rod configurations. Opt. Laser Technol..

[30] Miyazaki H.T., Mano T., Kasaya T., Osato H., Watanabe K., Sugimoto Y., Kawazu T., Arai Y., Shigetou A., Ochiai T. (2020). Synchronously wired infrared antennas for resonant single-quantum-well photodetection up to room temperature. Nat. Commun..

[31] Ogawa S., Kimata M. (2017). Wavelength- or Polarization-Selective Thermal Infrared Detectors for Multi-Color or Polarimetric Imaging Using Plasmonics and Metamaterials. Materials.

[32] Chen C., Huang Y., Wu K., Bifano T.G., Anderson S.W., Zhao X., Zhang X. (2020). Polarization insensitive, metamaterial absorber-enhanced long-wave infrared detector. Opt. Express.

[33] Tan X., Zhang H., Li J., Wan H., Guo Q., Zhu H., Liu H., Yi F. (2020). Non-dispersive infrared multi-gas sensing via nanoantenna integrated narrowband detectors. Nat. Commun..

[34] Pustelny T., Struk P., Mergo P., Wojtas J., Martyniuk P., Kopytko M., Grodecki K., Gawron W., Gomuka E. High operating temperature long-wave HgCdTe detector for fast response operation: Optimization approach. Proceedings of the 11th Conference on Integrated Optics: Sensors, Sensing Structures, and Methods.

[35] Forrest W. (2012). Development of Long-Wave HgCdTe Detector Arrays for Future Space Missions. NASA APRA Propos..

[36] Hu W., Ye Z., Liao L., Chen H., Lu W. (2014). A 128×128 long-wavelength/mid-wavelength two-color HgCdTe infrared focal plane array detector with ultra-low spectral crosstalk. Opt. Lett..

[37] Ehsan A., Mahsa K.S., Amir A., Andrei F. (2018). Full Stokes imaging polarimetry using dielectric metasurfaces. ACS Photonics.

[38] Sen S., Rhiger D.R., Curtis C.R., Kalisher M.H., Hettich H.L., Currie M.C. (2001). Infrared absorption behavior in CdZnTe substrates. J. Electron. Mater..

