# Supplementary material for: Design of Long-Wave Fully Polarized HgCdTe Photodetector Based on Silicon Metasurface
Source: Micromachines (Basel). 2025 Aug 14;16(8):937. doi: 10.3390/mi16080937 (PMC12388148; doi:10.3390/mi16080937)
Supplement: Supplementary file 1 [file micromachines-16-00937-s001.zip › micromachines-3760281-supplementary.pdf]

The angular dispersion characteristics of the metasurface are also an important indicator of the polarization detector. Figure S1(a) shows the influence of the incident angle on the polarization detector when the incident plane is the XZ plane. It can be found that when the incident angle increases from 0 degrees to 8 degrees, the absorption peak of LCP and the absorption valley of RCP simultaneously redshift. This redshift phenomenon of the central wavelength indicates that the incident angle can be inferred by monitoring the wavelength of the detector. However, as shown in Figure S1(b), when the incident light is in the YZ plane, the incident angle is almost independent of the absorption spectrum of the detector, which means that the detector may have a relatively high tolerance for the incident angle. In general, the change in the incident angle does not cause the polarization selection ability of the polarization detector to disappear, but it has a significant impact on the absorption wavelength of the detector.

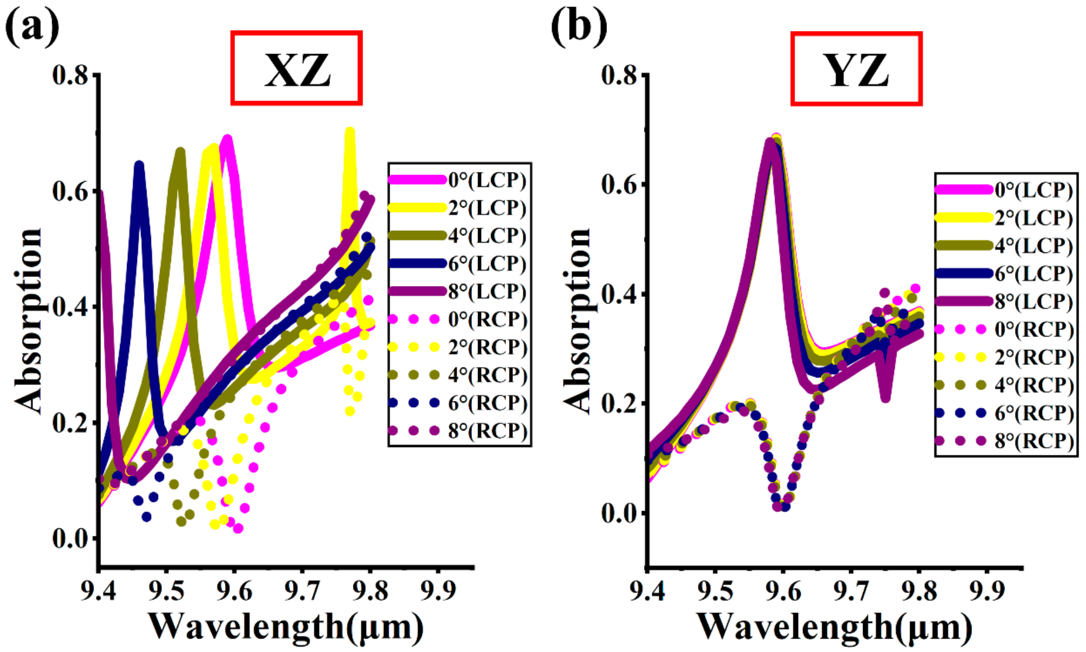

Figure S1. The influence of the incident angle. (a) The incident plane is the XZ plane. (b) The incident plane is the YZ plane.

The MgF_2 film has temperature dependence. Generally speaking, the variation of temperature will cause changes in both the thickness and refractive index of the spacer layer MgF_2. Figure S2(a) shows the influence of the thickness variation Δhs of the spacer layer. It can be found that the absorption spectrum of the circularly polarized detector is almost independent of Δhs, which also indicates that the device has a very high tolerance to Δhs. As shown in Figure S2(b), as the refractive index variation Δn of the spacer layer increases from -0.1 to 0.1, the working wavelength of the device redshifts from 9.55 μm to 9.66 μm. This means that the working wavelength of the device can be regulated by changing the temperature.

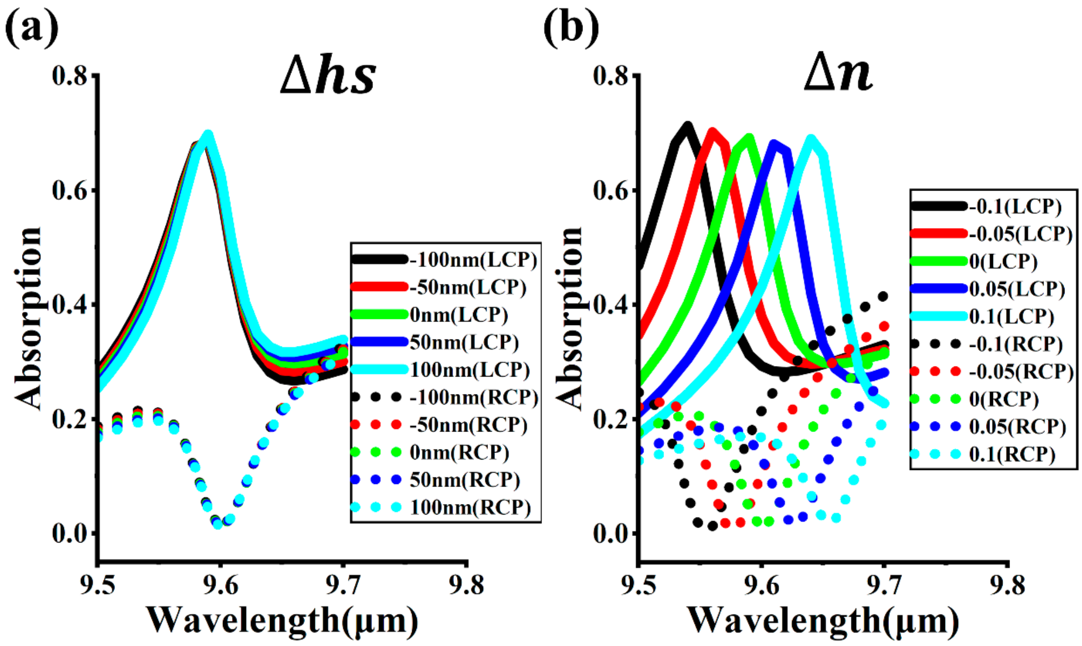

Figure S2. The influence of the changes in thickness and refractive index of the spacer layer caused by the temperature effect. (a) The variation in the thickness of the spacer layer Δhs. (b) The variation in the refractive index of the spacer layer Δn.

The specific manufacturing/characterization content of the proof-of-concept is as follows. First, the HgCdTe absorption layer is epitaxially grown on the CdZnTe substrate, and its absorption region is defined through the standard HgCdTe infrared detector process to form a mesa structure; then, a MgF_2 layer is deposited as a mechanical support and optical structure layer, and after depositing Si, a silicon metasurface with subwavelength structures is fabricated on the MgF_2 surface using electron beam lithography and other techniques to form independent resonant units; finally, a metallization process is used to form the top electrode of each pixel (D1-D6) and the common bottom electrode, and the chip-level packaging is completed. During the measurement, an infrared light is emitted by a blackbody radiation source, which is focused onto the surface of the detector array by an objective lens. A bias voltage is applied through a probe station, and the voltage response signals of each pixel are simultaneously read to characterize the device performance.

In addition, a thermoelectric cooler (TEC) is a thermoelectric cooling device based on the Peltier effect, which can achieve active temperature control by adjusting the current direction. In this polarization detector, perhaps a TEC (such as a multi-stage cooling module) can be installed beneath the MCT detector and coupled tightly with the detector chip through a high thermal conductivity material (such as an oxygen-free copper heat sink or a diamond heat spreader) to ensure efficient heat conduction and stabilize the operating temperature of the MCT. The advantages of this combination are: 1) suppressing the dark current and thermal noise of the MCT device, thereby enhancing the detection sensitivity; 2) achieving temperature stability through a closed-loop

temperature control circuit, ensuring the consistency of the metasurface optical performance and the MCT response; 3) being more compact than liquid nitrogen cooling, making it suitable for miniaturized integrated systems. This new cooling technology is expected to ensure the accuracy of polarization detection while considering miniaturization and reliability, especially suitable for long-wave infrared polarization imaging applications in field or airborne environments.

The optical model in the manuscript was developed based on COMSOL 5.6 version, and the corresponding physical module is the Wave Optics module. The optical model is a three-dimensional model with six end faces. Since the metasurface is periodic in the transverse direction, periodic boundary conditions can be applied to the four end faces in the X and Y axes. In addition, the two end faces in the Z-axis direction correspond to the Perfectly Matched Layer, which can eliminate the interference of simulation boundary reflection. The meshing strategy of the model is as follows:

Table S1： Meshing strategy of the model

Materials	Air	CdZnTe	Si	MgF_2	MCT
Mesh size（nm）	$\lambda_0/8$	$\lambda_0/8/2.7$	180 nm	$\lambda_0/8/3$	200nm
